# Restorative justice as customized creativity: Tinker Bell’s magic

**DOI:** 10.3389/fsoc.2023.1220470

**Published:** 2024-01-11

**Authors:** Bonnie Talbert

**Affiliations:** Committee on Degrees in Social Studies, Harvard University, Cambridge, MA, United States

**Keywords:** philosophy, boredom, restorative justice, creativity, cultural criminology

## Abstract

While many scholars have noted a rise in boredom coinciding with the emergence of modern capitalism, philosophers have long maintained that boredom is part of the horizon of human experience. Although specific social conditions may exacerbate it, boredom will never be completely eradicated. Nevertheless, its presence indicates that something is not right. Recently, cultural criminology has highlighted that boredom and monotony can trigger criminal behavior. If boredom is a contributing factor to crime, then I propose that creative, restorative justice processes can serve as an effective antidote. These practices aim to make things right by establishing obligations that restore the dignity and meaning of a victim’s life.

## Introduction

It would be natural to see creativity and justice as contradictory goals. Creativity is often associated with characteristics like uniqueness and spontaneity, whereas modern systems of justice often strive for equality under the law such that everyone is subject to the same rules. But there are compelling reasons to think that creative justice is no oxymoron, and may in fact provide a more satisfying and lasting path toward fairness, equity, and repair. In this essay, I will examine some creative, restorative solutions to crime that have opened up new possibilities for thinking about the relationship between crime, creativity, and restorative justice.

Inspired by Indigenous practices, the idea of restorative justice emerged in the United States as an alternative to the Western legal system (Zehr, 2015a), and aspires to make good on the case-by-case tradition that favors particularities over more uniform procedures. Like creative projects in general, restorative justice focuses on particularities and considers a range of possible combinations to achieve satisfying results. Within the constraints of the possible – when human lives cannot be revived, or funds cannot recompense financial losses, restorative justice explores satisfaction for victims that is often more symbolic than material. An interesting and exemplary case is known as “Tinker Bell,” named for the artwork commissioned by a victim from her victimizer. The deliberations and the realization of the work illustrate ways in which creativity, connection, and victim satisfaction can be achieved at the same time as behavioral reform to forestall future harm.

Harmful behavior, in this and many other cases, has a trigger in the under-examined danger of boredom, according to scholars in the field of cultural criminology. And so the remedy for wrongdoing must address this often unsuspected cause of crime: a compulsion to relieve boredom. To appreciate the trigger and the resolution in the Tinker Bell and other such cases, an examination of boredom will be instructive.

## Boredom: beyond modernity

The history of the philosophy of boredom is rich and fascinating if underappreciated (Toohey, 2012; Koerth-Baker, 2016). While the COVID-19 pandemic reinvigorated both scholarly and public interest in exploring the nature and significance of boredom, the topic has long been a philosophical and practical concern.

Boredom is frequently conceptualized as a consequence of the development of commercial society. Many scholars in cultural studies claim that boredom is a uniquely contemporary phenomenon (Dalle Pezze and Salzani, 2009). These arguments suggest that our modern lives and monotonous jobs are, in large part, what make us bored. Within his own time, Adam Smith, known for formulating the logic of capitalism, famously wrote that the division of labor contributes to the boredom of workers by requiring them to do the same thing over and over. As a consequence, Smith (1981) added, bored workers become incapable of breaking out of their mind-numbing activities, i.e., their boredom makes them boring (839–840). Even worse, it is not just a few workers on the assembly line who are negatively affected by the division of labor; the majority of people in commercial society are targets for boredom, resulting in a collective loss of creativity and imagination (840).

While Smith rightly noted that the division of labor and constant repetitive work can produce boredom, the origins and manifestations of boredom are not confined to contexts of commercial society. Boredom has been a part of the human experience long before the rise of contemporary job structures (Svendsen, 2005; Kuhn, 2017; Ros Velasco, 2017). Even in the most exciting jobs and favorable social conditions, people can feel bored. This inherent and ubiquitous nature of boredom underscores its existential roots, affirming its persistence across varied socio-cultural and occupational landscapes.

Yet, even if boredom is a standard part of human experience, this does not mean we should welcome it. Many other philosophers (Schopenhauer, 1969; Nietzsche, 1993; Pascal and Krailsheimer, 1995; Kierkegaard, 1998; Heidegger, 2019; Sartre, 2021) have suggested that boredom is both an existential part of the human condition as well as something we should challenge.

## Boredom: a brief conceptual analysis

Boredom is often understood as a complex, subjective experience that affects each person differently. Some are more distressed by it than others, and some are bored by things others find captivating. At the same time, almost no one gets to live a life without moments of boredom. Similar to feelings like pain or sadness, boredom signals that something is amiss. This is where boredom stands apart from ‘downtime’ or ‘rest.’ Unlike boredom, periods of rest are welcomed as beneficial, as contributing to creativity and increased productivity, and as a necessary break from the busy nature of daily work life (Gump and Matthews, 2000; Baumeister and Tierney, 2011; Zomorodi, 2017). While both boredom and rest involve stepping back from active engagement, they have distinct implications; boredom indicates a problem or misalignment in one’s mental state, while downtime is either neutral or refers to a positive, rejuvenating experience.

In his analysis of boredom, the philosopher Harry Frankfurt (1992) characterizes boredom as a state where an individual feels detached or disconnected from their desires. On this view, when we are bored, we are not simply in a state of not having anything to do but rather in a state where we find ourselves unable to connect with or care about our interests. This leads to what he calls an “attenuation of psychic liveliness,” where a person feels internally “deadened” or “flat.” When people are bored, they do not feel compelled or moved by any particular desire, interest, or concern.

This concept of boredom is part of Frankfurt’s broader philosophical account of the structure of human desires. He famously differentiates between “first-order” desires (desires for various things or outcomes) and “second-order” desires (desires about desires, such as wanting to want something). For Frankfurt, boredom can be seen as a failure or disconnection at this second-order level. When we are bored, it is not that we do not have first-order desires (such as desires to read a book or go for a walk); it is that we lack second-order volitional engagement with those desires. We might know things we could or should want to do, but we do not feel engaged or connected to those wants. Thus, the “attenuation of psychic liveliness” in boredom is not about the absence of things to do but the more profound disconnection or detachment from our motivational structures. This understanding of boredom reveals that there is more to the common perception of boredom as mere idle inactivity. As the Russian classic writer Tolstoy (2013) described, the state of boredom is “a desire for desires.”

While Frankfurt does not offer a prescriptive guide on how to live our lives, his insights on boredom provide several reasons to resist or confront it. Because boredom signals a detachment from our desires, resisting boredom can be seen as an effort to reconnect with what we genuinely care about or value. Pushing back against boredom becomes an endeavor to revive or maintain our inner vitality and engagement with the world. We can interpret boredom as a signal that indicates that it is time to reinfuse our lives with meaningful activity, ensuring that our lives are adequately directed toward things we care about. Frankfurt suggests that the human desire to avoid boredom is a basic instinct, not just because boredom is unpleasant, but because it dulls our mental processes. When we are bored, our attention and responsiveness to what is happening around us decreases, and we fail to notice or make important distinctions, leading to a more uniform and less varied conscious experience: “…[T]he avoidance of boredom is a very fundamental human urge. It is not a matter merely of distaste for a rather unpleasant state of consciousness. Being bored entails a reduction of attention; our responsiveness to conscious stimuli flattens out and shrinks; distinctions are not noticed and not made, so that the conscious field becomes increasingly homogeneous. The general functioning of the mind diminishes,” (12). In its extreme form, this lack of differentiation in our consciousness can lead to a state that is almost equivalent to having no conscious experiences at all: “Its tendency is to approach a complete cessation of significant differentiation within consciousness; and this homogenization is, at the limit, tantamount to the cessation of conscious experience altogether,” (12).

Various strategies have been employed to resist or alleviate boredom, some leading to unconventional and even destructive paths for escape. Several scholars have presented the idea that crime, as unexpected as it may sound, could serve as one of these outlets. Frankl (2006), a renowned psychologist, and Holocaust survivor, provides an illuminating perspective on this phenomenon. He asserted that, “…[T]he place of frustrated will to meaning is taken by the will to pleasure,” (79). The void left by meaninglessness, what Frankl calls an existential vacuum, leads to a state of boredom where individuals can feel pulled by the allure of transient and often superficial pleasures, feelings that can be associated with crime. The immediacy of the gratification provided by these pleasures serves as a temporary escape from the overbearing sense of emptiness.

## Boredom and cultural criminology: resistance to rationalized control

In 1988, Jack Katz introduced his phenomenological approach to crime, which emphasized the immediate “foreground” motivations, such as the immediate emotional experiences of crime, and, in contrast to traditional criminology, focused less on background conditions such as race, class, and gender (Katz, 1988). This approach to crime centers the lived reality of the perspective of those involved, with attention to the meanings, feelings, and understandings that characterize the first person experience of crime. Looking beyond objective and structural explanations, a phenomenological perspective captures the thrill, risk, pleasure, or fear that individuals might experience during criminal acts.

Building on Katz’s work, Jeff Ferrell’s perspective on crime centers the socio-cultural and experiential motivations behind criminal acts.

Ferrell (2007, 293) suggests that some crimes are motivated not by a desire to harm others or their property but by an attempt to escape boredom.

According to Ferrell, many modern routines and regulations can lead to a pervasive sense of boredom, such that the act of committing a crime can be seen as a form of symbolic interaction: a way for individuals to express discontent, resist cultural norms, and seek excitement amidst a monotonous societal backdrop. Crimes committed out of boredom are often expressive, countercultural acts that subvert the repetitive dullness of modern life: “Excitement, it seems, is in reality a means to an end, a subset of what ultimately emerges as the antidote to modern boredom: human engagement,” (294).

Ferrell’s concept is partly inspired by Raoul Vaneigem’s Situationist Critique of contemporary Western societies, which identifies increased boredom as one of the most dreadful aspects of modern life. Vaneigem warns of the dangers of intense boredom, stating, “Anyone who has felt the drive to self-destruction welling up inside him knows with what weary negligence he might one day happen to kill the organisers of his boredom… For passion destroyed is reborn in the passion for destruction” (Vaneigem and Nicholson-Smith, 2012, 162).

In considering the link between boredom and destructive behavior, Ferrell and other cultural criminologists have pointed out that identity is often linked to consumption in consumerist societies. Those who cannot participate in this consumer culture due to socio-economic constraints might feel left out or devalued. Engaging in crimes like theft or vandalism can be a form of reclaiming agency and challenging this consumerist paradigm. For those who feel alienated or marginalized, crime can offer a narrative or identity that breaks away from the perceived dullness and invisibility of everyday existence.

It is important to note that it is not just people who are found guilty of crimes that feel the pull to resist the monotony and rationalized control that modernity is said to wield. Ferrell explains: “…[t]he criminal, the consumer and the cultural revolutionary are perhaps more alike than different—that for them boredom creates a certain vacant commonality. After all, desperately looking for life amid boredom’s deteriorating death, the line between pleasure and pain, between crime and commodity, can be a thin one indeed,” (Ferrell, 2007, 294).

Ferrell’s perspective on crime and boredom is similar to the idea of edgework, a concept introduced by Lyng (2004), that refers to voluntary risk-taking activities that push participants to the boundaries or “edges” of their emotional, psychological, and physical limits. These activities often offer a thrill or a sense of challenge as people confront their fears. Examples of edgework activities include extreme sports, high-stakes gambling, certain forms of illicit drug use, and some criminal activities. Edgework is not just about taking risks; it is also about mastering or controlling those risks. Just as a skydiver seeks to control the risk of jumping out of an airplane, a person involved in certain criminal activities might take pride in their ability to outsmart law enforcement, navigate dangerous situations, or maintain composure under pressure. Other scholars such as Hayward and Young (2004) similarly encourage criminologists to examine the intense feelings associated with crime, such as the anger, humiliation, exuberance, excitement, and fear that are present throughout the whole process, from “the intense gutted feelings of the victim, to the thrill of the car chase, to the drama of the dock, to the trauma of imprisonment,” (264).

Steinmetz et al. (2017) found that boredom is a unifying experience across disparate criminological populations, such as detectives, prisoners serving life sentences, and hackers. They suggest that crime and deviance are linked to stifling social conditions that produce stunted identities and offer few opportunities for personal transformation and character development. They have also shown that even less exciting forms of crime and deviance are “linked to the same circumstances that contribute to spectacles of violence or the seemingly reckless displays of skill and bravado involved in edgework” (355).

Given this body of research, it seems plausible that a desire for excitement and creativity is a deep psychological need and an antidote to boredom. If we view crime as (at least in part) a reaction to boredom, we should consider practices and policies that address the features of boredom, such as lack of meaning, agency, creativity, and energy. Finding responses to crime that recognize and incorporate the psychological need for engagement, inventiveness, and spontaneity without causing undue harm to others and perpetuating cycles of violence is thus crucial in seeking successful and meaningful justice.

One way to address this need is to consider creative restorative justice practices, which under the right conditions, can provide a potential solution to the boredom that can lead to crime. Promoting human engagement, insight, creativity, and meaning, restorative justice can itself offer a rebellion against isolation and boredom.

## Defining creativity

A full analysis of creativity is beyond the scope of this essay, but briefly it is useful to think about creativity in terms a *process* that allows for a creative result, rather than focusing solely on the result. Many accounts of creativity maintain that in order for a product to count as creative, it must be brought about in the right way. Random accidents or mistakes can result in something surprising and new, but we would not typically call such results creative. The wind might blow a unique image into sand on the beach, but we would not think of that as the product of creativity. Typically, we think of creativity as a process that requires spontaneity, i.e., not every step in a creative process can be planned out in advance. As Gaut (2018, 133–137) points out, spontaneity comes in degrees. Plans to create something can be more or less developed, and it can be the process, the result, or both that are unknown at the start. In this sense, a kind of epistemological ignorance, or not-knowing is part of the creative process.

Creativity is also thought to be something that allows for originality. As Kronfeldner (2009) uses the term ‘original’, it does not simply mean producing something ‘new.’ Two people can independently come up with the same creative solution to a problem, for example, and as long as one did not copy from another then the discovery can still be original for each person even if they did not discover it first. Kronfeldner’s account of creativity differentiates between ‘historical’ novelty and ‘psychological’ novelty. It suggests that from a psychological perspective, a person can be deemed creative even if the idea or artifact they produce is not the first of its kind in history. For example, she notes that a potter who independently develops a way of making pots that resembles traditional methods, without having been influenced by them, can be considered creative. This is because the creation is psychologically new to the potter—it originated from their own thought processes and was not imitated. The main idea behind this distinction is that psychological creativity is concerned with the individual’s experience and process of creation rather than the uniqueness of the product in the broader historical context.

These two elements of creative processes, spontaneity and originality, I argue, can be part of restorative processes insofar as they allow for creative results. So if we see crime as a response to boredom, then a creative response to crime holds promise as one way to effectively address the fundamental problem.

## Restorative justice, creativity, and Tinker Bell

Restorative justice is a quickly growing field that has become central to discussions of harm, crime, punishment, and power. Yet it is not always obvious what the core ideas and practices of restorative justice are. According to Howard Zehr, who started the first formal restorative justice program in the United States in 1978 in Indiana, restorative justice is “a process to involve, to the extent possible, those who have a stake in a specific offense and to collectively identify and address harms needs and obligations in order to heal and put things as right as possible,” (Zehr, 2015a, chap. 2).

Restorative justice is based on a philosophy that focuses on repairing human relationships. In a restorative framework, when a crime occurs, the primary obligation is to do right by the people harmed to the extent possible. Restorative justice seeks solutions that repair harm by facilitating dialogue between the victim, the responsible party, and other affected parties.

In contrast to a “retributive justice” framework, where crime is an offense against the state, and the questions are centered around what crime was committed and how it should be punished, a restorative justice framework asks who was harmed, what they need, and whose obligation is to meet those needs. Crime is viewed as a violation of people and relationships rather than primarily as a violation of policy, and a crucial step in the restorative journey is to strengthen those relationships, which means creating spaces for people to have important discussions. This is often done in facilitated “circles” where people can tell their stories and work with others to devise a plan for how to move forward together. Because there is no singular way to repair a relationship, and because every victim needs something different, restorative justice creates conditions for individualized and creative solutions to crime.

These circles are often places to practice creative justice because there is no predetermined outcome. There is no singular solution that works for every case, or even for cases of a similar type. What each survivor needs is different and what each person who caused harm can do to take accountability for their actions is different. The process is necessarily spontaneous, because in most cases, while there are guidelines for best practices that participants follow, neither the details of how the process will go nor the end result can be known in advance. In most cases, processes will also be original, at least in the psychological sense, because the individuals’ experiences and actions in the process of creating restorative communities and agreements cannot easily be copied.

In a notable example of the creative power of restorative justice, sujatha baliga tells the story of a young man who had stolen a woman’s car (Butler and Butler, 2018). The victim worked in law enforcement and did not seem eager to work with the young man. When asked what would make things right, she replied that the cost of her car was the only thing that would suffice. Knowing the young man’s family was struggling financially, baliga brought the two parties together for a conversation, and after a long discussion, the woman asked for a life-sized Tinker Bell painting as restitution. “Tinker Bell. A Tinker Bell as big as me,” the woman requested. “And not the new one. The original Tinker Bell. I will forgive you the debt if you paint me a Tinker Bell as big as me.” The young man worked with a local artist and painted a Tinker Bell for the victim to put on her wall. As a result, relationships were restored. The young man stopped stealing cars and began working with the art organization he partnered with as he made Tinker Bell, and ultimately found meaningful, creative work there.

Sered (2019, 148) provides other examples of restorative justice processes’ creative and meaning-making power. In one instance, after an immigrant was robbed, the circle agreements included fairly standard practices such as apologies, community service, and education. During the restorative process, the person who robbed the immigrant explained that all the older men in his family had been to prison, and his older brother had won a prison boxing league championship. Since his brother taught him to defend himself, he offered to show his victim how to box as a way to make sure that he would not be afraid of being robbed again.

There are many more examples of innovative programs that have incorporated art and creativity into restorative justice process, demonstrating the potential for creative expression to facilitate rehabilitation and reconnection to both one’s own values and to one’s community (Walters, 2014). Young New Yorkers and Project Reset are two New York-based organizations that have developed successful diversionary programs that combine art and restorative justice.

Daniel Aguilar, who became an ambassador for Young New Yorkers after participating in the program as a teenager, has a compelling testimony about the transformative role art played in his life (Murali, 2020). Through creating collages and capturing videos, he engaged in deep self-exploration, asking fundamental questions about his identity and future. These activities provided him with a “safe space” to gain perspective and contemplate a larger purpose in life.

Project Reset collaborates with local arts institutions, such as the New Museum and the Brooklyn Museum to design meaningful art experiences for participants.

As part of Mural Arts Philadelphia, a program called The Guild offers former inmates the chance to participate in creative projects such as mural making, mosaics, and carpentry, while also offering job support and professional development workshops. Participants in The Guild show a lower tendency to reoffend, with recidivism rates below 15%, significantly below the state average of 35% (Mural Arts Philadelphia, n.d.).

Across the Atlantic, the Oxfordshire Youth Offending Service in the United Kingdom employs art as a reparative gesture towards victims or the community. To make amends, offenders make pictures, mosaics, or paintings to offer as gifts to their victims or to the community (Liebmann, 2007, 398). Clair Aldington, a supervisor at the service, observes, “I believe that for reparation to be successful as part of a restorative justice process it has to be meaningful for the young person, as well as for the victim (s) of their offence. There is something powerful about working alongside a young person to develop a skill that enables them to value themselves in a new way and to begin to see the potential for change in their life,” (397).

Through these programs, art becomes more than a medium of expression—it becomes a catalyst for personal transformation, a renewal of psychic liveliness, and a way to subvert the tedium and dullness that are often precursors to criminal behavior. The value of these restorative acts lies not in their rationality or logic, although sometimes they are logical, but in their capacity to help victims and people who caused harm reintegrate and reconnect to life with other people.

Although not all restorative justice processes integrate significant elements of creativity, the examples briefly mentioned here can inspire us to consider the power we have to devise imaginative and meaningful solutions to some of the most complex and intractable conflicts we face. Restorative justice is not a panacea but directs our attention to what philosophers have urged us to focus on for centuries: meaning, creativity, collaboration, and spontaneity. We can never predict what people will come up with to solve a problem, but restorative processes can be exciting and empowering, providing new paths for the parties involved to reorient their lives toward new sources of meaning. Lower recidivism rates (Steiner and Johnson, 2003; Bergseth and Bouffard, 2007, 2012; Liebmann, 2007) and other benefits of restorative justice such as increased victim satisfaction (Steiner and Johnson, 2003, 55) are significant, but the way it empowers the people affected by crime to create something new offers an antidote to not only to crime but also to boredom. As sujatha baliga teaches us, “You can never really predict what will make a victim whole. Sometimes, it’s Tinker Bell.”

While advocating for restorative justice, Zehr acknowledges that it is not a cure-all solution. He encourages skepticism and a critical perspective, stating, “I’d rather you be a skeptic than a true believer.… I want people to have a mixture of criticism and advocacy for it,” (Zehr, 2015b). As part of restorative justice work, Zehr emphasizes the importance of acknowledging actual and potential issues and limitations. This approach allows for growth and evolution in the field as well as room for creativity and new ideas to emerge.

It is important to note that there is still much that is unknown about the directions that restorative justice might take, which can naturally lead to healthy skepticism. However, this also leaves room for hope and optimism about the future of restorative justice. By recognizing and incorporating the psychological need for creativity, novelty, and spontaneity, restorative justice offers a means of rebellion against boredom and a path toward insight, creativity, and meaning through human engagement. While it is crucial to remember that restorative practices will not solve problems of conflict, harm, and crime once and for all, we should also keep in mind that they can offer an antidote, sometimes in the form of Tinker Bell, to traditional punitive approaches. Restorative justice engages, connects, and encourages participants to actively create a unique path forward.

## Author contributions

The author confirms being the sole contributor of this work and has approved it for publication.
